# Trends in research on fatigue in rheumatology: Bibliometric analysis

**DOI:** 10.1515/rir-2026-0019

**Published:** 2026-07-13

**Authors:** Zulal Tatar, Elif Gur Kabul, Bilge Basakci Calik

**Affiliations:** Department of Physiotherapy and Rehabilitation, Faculty of Health Sciences, Inonu University, Malatya, Türkiye; Department of Physiotherapy and Rehabilitation, Faculty of Health Sciences, Usak University, Usak, Türkiye; Faculty of Physiotherapy and Rehabilitation, Pamukkale University, Denizli, Türkiye

**Keywords:** rheumatology, fatigue, rheumatic disease

## Abstract

**Objective:**

The aim of this study is to analyze the studies on fatigue symptoms in rheumatology using bibliometric methods and to determine current trends and focal points.

**Methods:**

The dataset for this study was created from articles published between 1983 and 2024 accessed *via* the Web of Science database. The keyword index used for WoS was determined as (TI = [“fatigue”] ).

**Results:**

A total of 624 articles were included in the study. The highest publication productivity was seen in the United States with 186 publications. The journals “Journal of Rheumatology” and “Rheumatology” both ranked first in publication productivity with 68 articles each. The most commonly used keyword was “rheumatoid arthritis”. The journal with the highest total citation amount and cumulative publication production was “Journal of Rheumatology”. Trending topics were seen tofacitinib, patient-reported outcome measures, and psoritic arthritis. Authors with one publication on fatigue in rheumatic diseases constituted 79% of all authors, authors with two publications constituted 12%, and authors with three or more publications constituted 4%. The most globally cited study was Wolfe, F. *et al*. (1996). The most common collaborating author keyword was “Rheumatoid Arthritis”.

**Conclusion:**

The literature on fatigue in the field of rheumatology contains deficiencies in better understanding the pathophysiology of fatigue, its management and treatment. At the same time, in recent years, fatigue has moved away from parameters such as muscle strength, pain and disease activity and towards patient reported outcomes. The increase in studies determining the validity and reliability of fatigue questionnaires in rheumatic diseases is also striking.

## Introduction

Fatigue is a highly prevalent and disabling problem among individuals with rheumatic diseases, presenting substantial challenges in clinical management and exerting a profound impact on quality of life.^[1]^ More than half of individuals with rheumatic diseases report experiencing severe fatigue.^[2]^ Despite its prevalence and clinical significance, patients frequently indicate that fatigue receives limited attention during medical consultations, as it is often not recognized as a primary symptom by many health professionals.^[3]^ Although advances in therapeutic strategies have led to effective control of disease activity, fatigue remains a persistent complaint during the course of treatment.^[4]^ Consequently, fatigue in rheumatic diseases represents a critical symptom warranting further investigation, both to elucidate underlying pathophysiological mechanisms and to inform the development of novel therapeutic approaches.^[5]^

Bibliometric analyses are employed to reveal emerging trends in article and journal performance, collaboration patterns, and research components, as well as to assess productivity within the existing body of literature and to identify gaps in the field.^[6]^ In recent years, bibliometric analysis has increasingly been recognized as a valuable methodology for evaluating scientific output.^[7]^

Fatigue is influenced by a wide range of physical, psychological, and social factors.^[8]^ Moreover, it is highly prevalent across various rheumatic diseases, including rheumatoid arthritis, ankylosing spondylitis, systemic lupus erythematosus, Sjögren’s syndrome, systemic sclerosis, vasculitis, and Behçet’s syndrome, largely due to the complex interplay between immune alterations and fatigue mechanisms.^[9]^ This underscores the necessity of examining fatigue in rheumatic diseases across different disease groups and from multiple perspectives. Despite its clinical importance, there remains a paucity of evidence-based data in the literature regarding effective fatigue management strategies for healthcare professionals.^[10]^ Accordingly, a comprehensive understanding of the fatigue-related literature within rheumatology is essential to address these gaps.

Changes in trends in fatigue studies in rheumatology are important because they demonstrate how the position of fatigue in research and clinical practice in rheumatic diseases has evolved over time. These changes provide insights into the transformation of clinical approaches to fatigue in rheumatology, revealing that fatigue is now a significant symptom in many rheumatic diseases, not just specific ones, and is studied in conjunction with various parameters. Furthermore, by examining the literature on fatigue as a crucial symptom in detail, it provides information about gaps and trends in the literature, thus shedding light on future research.

The aim of the present study is to identify current trends and focal points in research on fatigue in rheumatic diseases through the application of bibliometric methods.

## Methods

### Study Design

A bibliometric analysis was conducted to elucidate current trends and focal areas within the literature concerning fatigue in rheumatic diseases. Bibliometric analysis constitutes a systematic review of scientific literature, enabling the identification of patterns, trends, and scholarly performance within a specific domain.^[11]^ Moreover, it facilitates the tracing of the field’s evolution and illuminates nascent and emerging research directions.^[6]^

### Data Sources and Search Strategies

In this study, the Web of Science (WoS) database was employed as the primary data source to investigate research on fatigue in rheumatic diseases through bibliometric methods. A comprehensive review of the relevant literature informed the selection of appropriate keywords. The keyword index used for WoS was defined as (TI = [“fatigue”] ).

**Figure 1:**
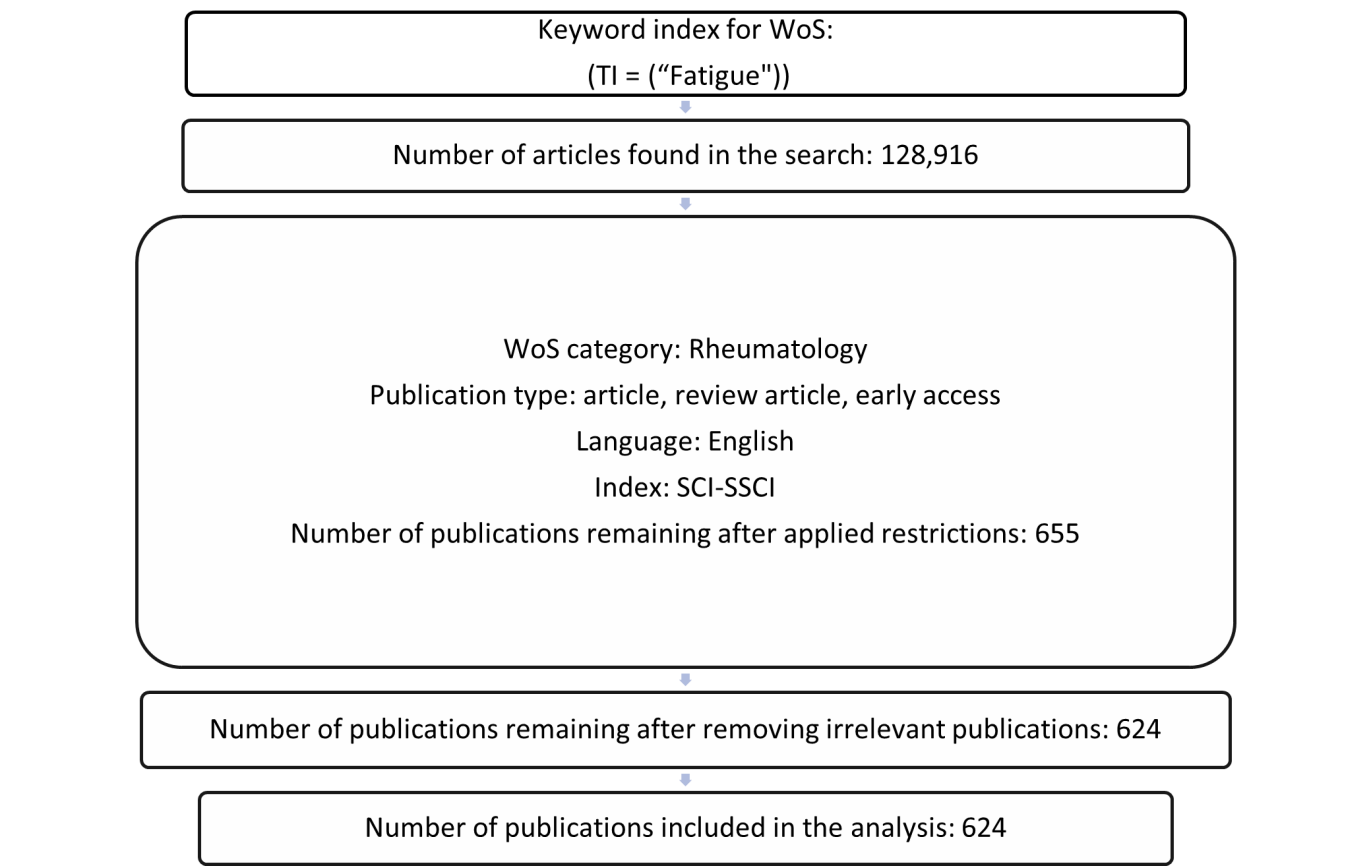
Flowchart of the study.

No restrictions were applied with respect to publication year. The WoS categories were limited to “Rheumatology. ” Original research articles, review articles, and early access articles were included as eligible WoS publication types, and English was chosen as the publication language. The literature search encompassed both the SCI and SSCI indexes. Titles and keywords of the retrieved articles were independently analyzed in detail by two researchers, and studies specifically addressing fatigue in rheumatology were included in the final dataset. A total of 31 off-topic studies were manually excluded (Figure 1).

### Data Analysis

Microsoft Excel 2016, VOSviewer 1.6.18, and the R bibliometrix package were utilized to conduct the bibliometric analyses. To illustrate the temporal development of the field, the distribution of publications by year was presented. Whereas publication metrics primarily assess the quantity of research output and the extent of collaboration, citation metrics aim to identify influential studies and emerging trends within a field by examining how publications are interconnected through citation networks. Moreover, combined metrics incorporating both citation counts and publication counts provide insights into the reception of research within the academic community, its overall impact, and the specific topics to which it contributes most significantly.^[12]^

To evaluate research performance, journal and publication productivity were assessed. The most influential journals and articles were identified through bibliometric analyses considering total publication counts, global citations, and local citations. Lotka’s law, which is concerned with determining author productivity, was employed to evaluate the productivity of researchers contributing to the literature on fatigue in rheumatic diseases.^[13]^

Science mapping techniques were further applied to examine the structure and dynamics of scientific research.^[14]^ Several approaches were employed: analysis of cited studies to identify the most influential publications; co-citation analysis to better understand the relationships among referenced works; co-word (common keyword) analysis to reveal connections among research topics; and co-author analysis to investigate social collaboration networks among researchers. Co-citation analysis identifies conceptual linkages and thematic similarities based on documents derived from shared references.^[15]^ Keywords serve as important indicators of frequently investigated areas within the field,^[16]^ and co-word analysis determines the co-occurrence of keywords, thereby clarifying relationships and illustrating the distribution of knowledge across different research domains.^[17]^ Co-author analysis focuses on elucidating patterns of scientific collaboration, providing collaboration statistics, and proposing valid and reliable criteria for identifying leading contributors.^[18]^ In addition, trending topic analyses were performed to determine emerging directions in the literature.

## Results

**Figure 2:**
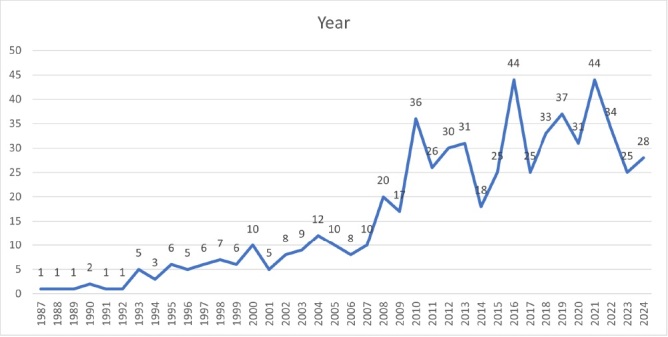
Publication productivity of the rheumatology by year.

When the distribution of 624 publications on fatigue in rheumatic diseases between 1987 and 2024 was analyzed by year, both increases and decreases were observed over time. While the number of publications rose steadily until 2008, fluctuations were noted thereafter. In particular, a decline in publication output was observed after 2021, followed by an increase again after 2023 (Figure 2). The years with the highest number of publications were 2016 and 2021, each with 44 publications. The most pronounced increase occurred between 2010 and 2011.

**Figure 3:**
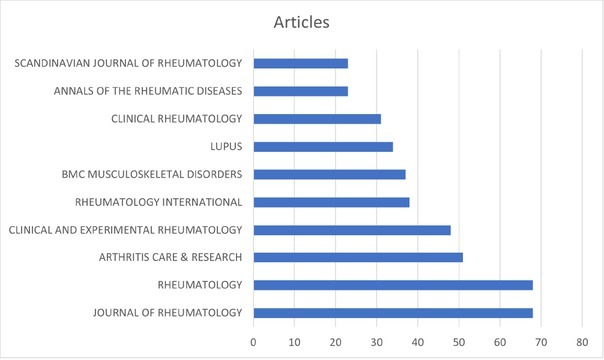
The top ten journals with the highest number of publications.

**Figure 4:**
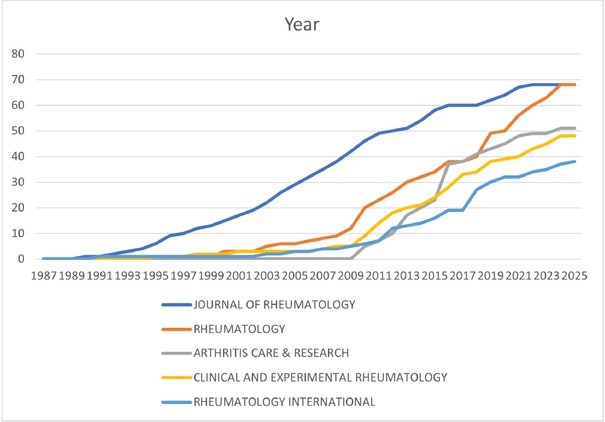
Cumulative number of publications by year for journals.

An analysis of journals publishing on fatigue in rheumatic diseases revealed that the most productive journals were The Journal of Rheumatology, Rheumatology, and Arthritis Care & Research, respectively (Figure 3). Examination of the cumulative publication output of these journals indicated a consistent increase in studies on fatigue in rheumatic diseases across all journals over the years. The Journal of Rheumatology demonstrated a stable trend in publication output during the past three years (Figure 4).

In terms of citation impact, the top three journals with the highest total citation counts were The Journal of Rheumatology, Rheumatology, and Arthritis Care & Research. The Journal of Rheumatology exhibited the highest h-index (h = 41), whereas Arthritis Care & Research recorded the highest m-index (m = 1.819) (Table 1).

**Table 1:** The H and M indices, total citations, and number of publications of the top ten journals with the highest publication count

Journals	H index	M index	TC	Articles
Journal Of Rheumatology	41	1.139	5343	68
Rheumatology	34	1.259	3397	68
Arthritis Care & Research	29	1.813	2178	51
Clinical And Experimental Rheumatology	19	0.613	1100	48
Lupus	19	0.731	880	34
Annals Of The Rheumatic Diseases	18	0.600	1658	23
Clınıcal Rheumatology	18	0.545	823	31
Rheumatology International	18	0.514	809	38
Bmc Musculoskeletal Disorders	17	0.773	840	37
Scandinavian Journal Of Rheumatology	17	0.436	633	23

TC = Total citiation.

**Figure 5:**
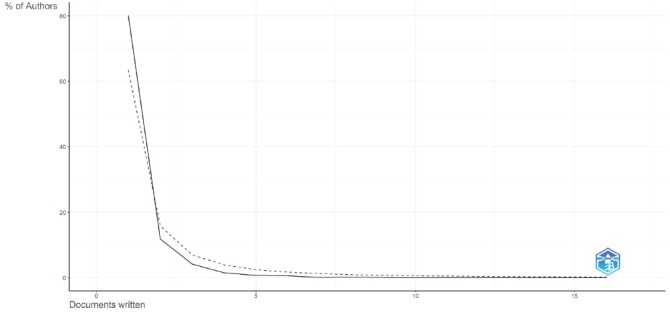
Author productivity through Lotka's Law.

According to Lotka’s law, regarding author productivity, 79% of authors in studies on fatigue in rheumatic diseases have published a single article, 12% have published two articles, and 4% have published three or more articles (Figure 5).

In terms of keyword frequency within fatigue research in rheumatic diseases, the most commonly used keywords were rheumatoid arthritis (*n* = 13), quality of life (*n* = 73), and systemic lupus erythematosus (*n* = 73) (Table 2). Other frequently occurring keywords included pain, fibromyalgia, patient-reported outcomes, depression, disease activity, sleep quality, and Sjögren’s syndrome.

**Table 2:** The top ten most frequently used keywords

Keywords	Frequency
Rheumatoid arthritis	113
Quality of life	73
Systemic lupus erythematosus	73
Pain	51
Fibromyalgia	47
Patient reported outcomes	40
Depression	39
Disease activity	36
Sleep quality	33
Sjogren’s syndrome	28

**Figure 6:**
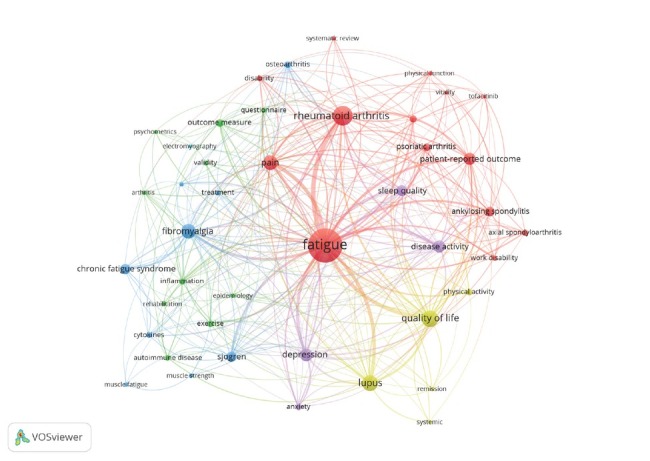
The common keyword clusters.

Common keyword analysis resulted in the identification of five distinct clusters (Figure 6). The red cluster represents the relative core of the network, indicating a higher frequency of research on these topics. This cluster comprised the keywords fatigue, rheumatoid arthritis, pain, ankylosing spondylitis, vitality, tofacitinib, disability, axial spondyloarthritis, work disability, patient-reported outcomes, physical function, systemic sclerosis, psoriatic arthritis, and systematic review. Several symptoms and factors associated with various rheumatic diseases were concentrated at the centers of this cluster. The purple cluster included keywords related to sleep quality, depression, anxiety, and disease activity. The blue cluster encompassed fibromyalgia, Sjögren’s syndrome, chronic fatigue syndrome, osteoarthritis, muscle strength and fatigue, cytokine markers, electromyography, and rheumatic disease. The green cluster contained autoimmune disease, rehabilitation, arthritis, epidemiology, outcome measures, exercise, psychometric properties, validity, scale, and inflammation. The yellow cluster comprised lupus, physical activity, quality of life, systemic involvement, and remission.

**Figure 7:**
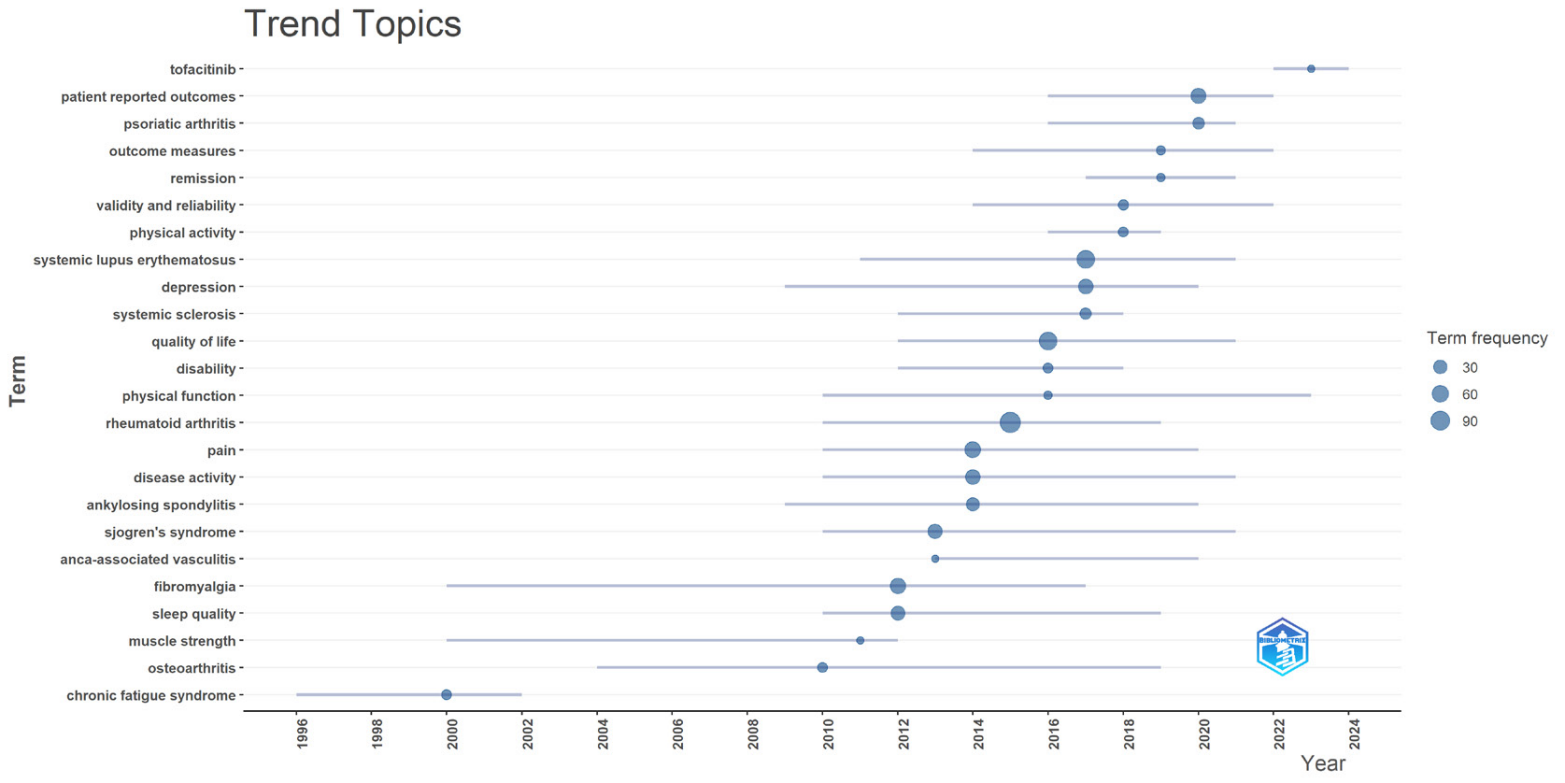
Trend topic.

In longitudinal analyses of fatigue research in rheumatic diseases, tofacitinib has emerged as a prominent topic in recent years. Patient-reported outcome measures have also gained notable attention. Psoriatic arthritis has been identified as a current focus among disease groups in fatigue research, whereas systemic lupus erythematosus represents a patient population that has been studied extensively over many years. Chronic fatigue syndrome, although frequently investigated in earlier studies, has declined in relevance in recent years (Figure 7).

The ten most globally cited articles in the field of fatigue in rheumatic diseases were analyzed. The studies most frequently cited locally were those by Wolfe *et al*. (1996)^[19]^, Cella *et al*. (2005)^[20]^, and Pollard *et al*. (2006)^[21]^, with average citations per year of 17.70, 19.57, and 16.15, respectively (Table 3).

**Table 3:** The global citations, citations per year, and normalized global citations of the top ten publications with the most global citations

Number	Publication	Global citations	Citations per year	Normalized global citations
1.	Wolfe F, Hawley DJ, Wilson K. The prevalence and meaning of fatigue in rheumatic disease. J Rheumatol. 1996;23:1407-1417^[19]^	531	17.70	4.02
2.	Cella D, Yount S, Sorensen M, Chartash E, Sengupta N, Grober J. Validation of the Functional Assessment of Chronic Illness Therapy Fatigue Scale relative to other instrumentation in patients with rheumatoid arthritis. J Rheumatol. 2005;32:811-819^[20]^	411	19.57	3.65
3.	Pollard LC, Choy EH, Gonzalez J, Khoshaba B, Scott DL. Fatigue in rheumatoid arthritis reflects pain, not disease activity. Rheumatology (Oxford). 2006;45:885-889^[21]^	323	16.15	3.02
4.	Hewlett S, Cockshott Z, Byron M, *et al*. Patients’ perceptions of fatigue in rheumatoid arthritis: overwhelming, uncontrollable, ignored. Arthritis Rheum. 2005;53:697-702^[22]^	322	15.33	2.86
5.	Belza BL. Comparison of self-reported fatigue in rheumatoid arthritis and controls. J Rheumatol. 1995;22:639-643^[23]^	299	9.65	3.23
6.	Dass S, Bowman SJ, Vital EM, *et al*. Reduction of fatigue in Sjögren syndrome with rituximab: results of a randomised, double-blind, placebo-controlled pilot study. Ann Rheum Dis. 2008;67:1541-1544^[24]^	287	15.94	3.89
7.	Kirwan JR, Minnock P, Adebajo A, *et al*. Patient perspective: fatigue as a recommended patient centered outcome measure in rheumatoid arthritis. J Rheumatol. 2007;34:1174-1177^[25]^	258	13.58	2.44
8.	Varni JW, Burwinkle TM, Szer IS. The PedsQL Multidimensional Fatigue Scale in pediatric rheumatology: reliability and validity. J Rheumatol. 2004;31:2494-500^[26]^	245	11.14	2.62
9.	Lentz MJ, Landis CA, Rothermel J, Shaver JL. Effects of selective slow wave sleep disruption on musculoskeletal pain and fatigue in middle aged women. J Rheumatol. 1999;26:1586-1892^[27]^	234	8.67	2.46
10.	Tench CM, McCurdie I, White PD, D’Cruz DP. The prevalence and associations of fatigue in systemic lupus erythematosus. Rheumatology (Oxford). 2000;39:1249-1254^[28]^	228	8.77	3.35

An analysis of country-level productivity revealed that the ten most productive countries were the United States, the United Kingdom, the Netherlands, Canada, Sweden, Türkiye, France, Germany, Norway, and Denmark (Table 4). The total number of publications for the top three countries was 186, 149, and 73, respectively. The United States also led in total citations, with 7228, and exhibited the highest average number of citations per publication at 58.20 (Table 5).

**Table 4:** Countries scientific production

Country	Number of Publications
USA	41
England	27
Australia	17
Netherlands	15
Canada	13

**Table 5:** Country productivity

Countries	TP	PT (%)	TC	AC
USA	186	29.808	7278	58.20
United Kingdom	149	23.878	4386	47.10
Netherlands	73	11.699	2284	41.50
Canada	69	11.058	2118	46.00
Sweden	42	6.731	830	37.70
Turkiye	42	6.731	857	23.20
France	41	6.571	874	31.20
Germany	36	5.769	402	33.50
Norway	36	5.769	1062	37.90
Denmark	26	4.167	635	35.30

TP: total number of published documents; PT (%): percentage of total published documents; TC: total number of citations; AC: average citations per publication.

## Discussion

This study analyzed research on fatigue within the field of rheumatology from 1987 to 2024 using bibliometric methods applied to scientific publications.

The total number of publications and their temporal trends provide valuable insights into research productivity within the field.^[29]^ Annual publication numbers showed a steady increase until 2009, after which there were fluctuations in publication numbers. This trend indicates that fatigue is increasingly accepted as an important symptom and outcome measure. Furthermore, the inclusion of fatigue as a key outcome measure for all future studies at the eighth OMERACT meeting in 2006 paved the way for increased research in this area.^[10,30]^ The periodic fluctuations in publication numbers show that fatigue research is a dynamic field in rheumatology, constantly evolving in parallel with changing clinical priorities. From a clinical perspective, fatigue requires a multidimensional assessment and close monitoring of current literature.

In the field of fatigue research in rheumatology, the journals with the highest publication output are The Journal of Rheumatology and Rheumatology. The long publication histories and broad scope of these journals may have contributed to this prominence. Older publications tend to receive more citations than newer publications, which can cloud temporal impact assessments.^[31]^ To complement this, the h-index and m-index were used to assess journal performance. Although Arthritis Care & Research shows relatively lower total citation numbers and publication numbers, its m-index surpasses that of other journals, indicating that its articles are consistently of high quality or currently have relatively high citation rates. These findings indicate that fatigue research in rheumatology is concentrated around certain core journals. From a clinical application perspective, studies published in journals with a high m-index have a higher potential to provide clinicians and researchers with current, effective, and applicable evidence.

The prominence of rheumatoid arthritis, systemic lupus erythematosus, fibromyalgia, and Sjögren’s syndrome as the most frequently used keywords indicates that the literature on fatigue is largely concentrated in these diseases. In this context, the concentration of the literature on specific rheumatic diseases leads to limited data on fatigue experiences in other rheumatic diseases and pediatric groups. This unbalanced distribution restricts the generalizability of the findings, potentially leading to incomplete or biased interpretations of the characteristics and management of fatigue in different disease groups and age populations.

The fact that quality of life, pain, sleep quality, disease activity, and depression are also frequently reported keywords suggests that factors related to these diseases are important focuses in fatigue research. Since fatigue is a subjective symptom,^[32]^ it explains the prominence of patient-reported outcome measures as a frequently used keyword. Fatigue research remains relatively limited in other rheumatic diseases and that the dimensions of fatigue should be further investigated in different rheumatological diagnoses in the future.

In recent years, fatigue research has shifted from traditional parameters such as muscle strength, pain, and disease activity to patient-reported outcomes reflecting the patient’s perspective. This trend reveals that fatigue in rheumatology should be evaluated not only with biomedical indicators but also in conjunction with the patient’s subjective experience. This has been reflected in an increase in studies examining the validity and reliability of fatigue questionnaires in rheumatic diseases. The fatigue is gaining importance as a patient-centered clinical outcome measure in rheumatology. In clinical practice, the systematic use of patient-reported outcome measures by rheumatologists and and physiotherapists in treatment planning and follow-up processes will contribute to the development of a more holistic and patient-centered approach.

An examination of trending topics reveals that tofacitinib has been prominent in recent studies. The EULAR 2023 systematic review reported that it reduces fatigue in psoriatic arthritis and rheumatoid arthritis.^[10]^ Tofacitinib reduces systemic inflammation, joint activity, and associated pain and morning stiffness by suppressing the signaling of numerous pro-inflammatory cytokines.^[33]^ Given that fatigue is closely related to these factors, it can be assumed that tofacitinib reduces fatigue secondarily, rather than directly, by controlling the underlying pathophysiological processes of the disease.^[34,35]^ For researchers, in particular, there is a limited focus on other interventions in fatigue-related research. This situation highlights the insufficient attention paid to pharmacological and non-pharmacological treatment strategies that directly target fatigue. However, this trend indicates that the effects of agents like tofacitinib, not only on disease activity but also on fatigue, can be considered in treatment selection.

Analysis of author productivity indicates that only 4% of research on fatigue in rheumatology has been conducted by a group of authors with three or more publications. This finding suggests that overall author productivity in the field is relatively low and that the number of researchers specialized in this area may be limited. The fact that the vast majority of authors have published only one study can be explained by the interdisciplinary nature of the field of fatigue in rheumatic diseases. This suggests that a core group of researchers producing consistent work has not yet formed and the field is still developing.

High global citation numbers indicate that an article has a significant impact not only in a specific field but also across multiple disciplines.^[6]^ The universality of fatigue as a symptom experienced worldwide may have contributed to the high number of interdisciplinary citations.^[36]^ Furthermore, the PedsQL Multidimensional Fatigue Scale, whose validity and reliability have been evaluated in pediatric rheumatology, is among the most cited studies. This information is clinically valuable. Clinicians should be aware that fatigue is also a significant problem in the pediatric rheumatological population.

Co-authored keywords capture the co-occurrence of terms and enable them to identify relationships between different research issues.^[11]^ The keywords rehabilitation and exercise have frequently been used together. Exercise is recognized as an effective and safe intervention for fatigue management in rheumatic diseases.^[37]^ Clinically, patients with rheumatic disease can be referred to a physiotherapist for safe exercise practices to manage their fatigue symptoms.

Although many countries have conducted research on fatigue in the field of rheumatology, some regions have not shown sufficient interest in this area. While most studies originate from America and Europe, African and Asian countries have shown limited participation in this field. The results may predominantly reflect data obtained from populations in these regions. This reduces the generalisability of the results and highlights the gap in the field. The United States being the most productive country can be explained by its large population and the high prevalence of rheumatic diseases associated with fatigue.

This study has several limitations. First, the bibliometric analysis was based exclusively on the WoS database, and other databases such as Scopus and PubMed were not included. Second, some relevant studies may have been omitted from the analysis if they were indexed under different WoS categories, potentially limiting the comprehensiveness of the findings.

## Conclusion

The field of fatigue in rheumatic diseases is still developing. Tofacitinib, not only on disease activity but also on fatigue, can be considered in treatment selection. Fatigue is also a significant problem in the pediatric rheumatological population. Patients with rheumatic disease can be referred to a physiotherapist for safe exercise practices to manage their fatigue symptoms. In clinical practice, to preserve functionality and reduce workforce loss, multidimensional factors such as fatigue, sleep quality, and psychological state can be considered together with structural damage.

## References

[1] Staud R (2012). Peripheral and central mechanisms of fatigue in inflammatory and noninflammatory rheumatic diseases. Curr Rheumatol Rep.

[2] Overman CL, Kool MB, Da Silva JA, Geenen R (2016). The prevalence of severe fatigue in rheumatic diseases: an international study. Clin Rheumatol.

[3] Connolly D, Fitzpatrick C, O’Toole L (2015). Impact of Fatigue in Rheumatic Diseases in the Work Environment: A Qualitative Study. Int J Environ Res Public Health.

[4] Korte SM, Straub RH (2019). Fatigue in inflammatory rheumatic disorders: pathophysiological mechanisms. Rheumatology.

[5] Seifert O, Baerwald C (2019). Impact of fatigue on rheumatic diseases. Best Pract Res Clin Rheumatol.

[6] Donthu N, Kumar S, Mukherjee D, Pandey N, Lim WM (2021). How to conduct a bibliometric analysis: An overview and guidelines. J Bus Res.

[7] Ellegaard O, Wallin J A (2015). The bibliometric analysis of scholarly production: How great is the impact?. Scientometrics.

[8] Louati K, Berenbaum F (2015). Fatigue in chronic inflammation - a link to pain pathways. Arthritis Res Ther.

[9] Sandıkçı SC, Özbalkan Z (2015). Fatigue in rheumatic diseases. Eur J Rheumatol.

[10] Farisogullari B, Santos EJF, Dures E, Geenen R, Machado PM (2023). Efficacy of pharmacological interventions: a systematic review informing the 2023 EULAR recommendations for the management of fatigue in people with inflammatory rheumatic and musculoskeletal diseases. RMD Open.

[11] Passas I (2024). Bibliometric analysis: the main steps. Encyclopedia.

[12] van Raan AFJ, Bockmans W, Engwall L, Weaire D (2014). Bibliometrics: Use and Abuse in the Review of Research Performance.

[13] Budd JM (1988). A bibliometric analysis of higher education literatüre. Res High Educ.

[14] Deng W, Liang Q, Li J, Wang W (2021). Science mapping: A bibliometric analysis of female entrepreneurship studies. Gend Manag: An Int J.

[15] Pessin VZ, Yamane LH, Siman RR (2022). Smart bibliometrics: an integrated method of science mapping and bibliometric analysis. Scientometrics.

[16] Çelik O T, Karaca M A (2024). Bibliometric profile of research on physical activities in special education. Int J Disabil Sports Health Sci.

[17] Hu K, Govindjee G, Tan J, Xia Q, Dai Z, Guo Y (2020). Co-author and co-cited reference network analysis for chlorophyll fluorescence research from 1991 to 2018. Photosynthetica.

[18] Uddin S, Hossain L, Abbasi A, Rasmussen K (2012). Trend and efficiency analysis of co-authorship network. Scientometrics.

[19] Wolfe F, Hawley DJ, Wilson K (1996). The prevalence and meaning of fatigue in rheumatic disease. J Rheumatol.

[20] Cella D, Yount S, Sorensen M, Chartash E, Sengupta N, Grober J (2005). Validation of the Functional Assessment of Chronic Illness Therapy Fatigue Scale relative to other instrumentation in patients with rheumatoid arthritis. J Rheumatol.

[21] Pollard LC, Choy EH, Gonzalez J (2006). Fatigue in rheumatoid arthritis reflects pain, not disease activity. Rheumatology (Oxford).

[22] Hewlett S, Cockshott Z, Byron M (2005). Patients’ perceptions of fatigue in rheumatoid arthritis: overwhelming, uncontrollable, ignored. Arthritis Rheum.

[23] Belza BL (1995). Comparison of self-reported fatigue in rheumatoid arthritis and controls. J Rheumatol.

[24] Dass S, Bowman SJ, Vital EM (2008). Reduction of fatigue in Sjögren syndrome with rituximab: results of a randomised, double-blind, placebo-controlled pilot study. Ann Rheum Dis.

[25] Kirwan JR, Minnock P, Adebajo A (2007). Patient perspective: fatigue as a recommended patient centered outcome measure in rheumatoid arthritis. J Rheumatol.

[26] Varni JW, Burwinkle TM, Szer IS (2004). The PedsQL Multidimensional Fatigue Scale in pediatric rheumatology: reliability and validity. J Rheumatol.

[27] Lentz MJ, Landis CA, Rothermel J, Shaver JL (1999). Effects of selective slow wave sleep disruption on musculoskeletal pain and fatigue in middle aged women. J Rheumatol.

[28] Tench CM, McCurdie I, White PD (2000). The prevalence and associations of fatigue in systemic lupus erythematosus. Rheumatology (Oxford).

[29] Mukherjee D, Lim W M, Kumar S, Donthu N (2022). Guidelines for advancing theory and practice through bibliometric research. J Bus Res.

[30] Hewlett S, Nicklin J, Treharne GJ (2008). Fatigue in musculoskeletal conditions. Topical Reviews: Rep RheDis Ser.

[31] Liu B, Liu S, Alastra AJ (2019). The 100 most cited vs most relevant articles in the journal of neurosurgery: a bibliometric analysis. Cureus.

[32] Redondo M, Leon L, Povedano FJ (2017). A bibliometric study of the scientific publications on patient-reported outcomes in rheumatology. Semin Arthritis Rheum.

[33] Nyberg L, Halfvarson J, Söderling J (2025). Prospective observational study of tofacitinib in ulcerative colitis–analysis of clinical data, fatigue and health-related quality of life during the induction phase. Ther Adv Gastroenterol.

[34] Kristensen LE, Navarro-Compán V, Magrey M (2023). Pain and Inflammation as Mediators of Tofacitinib Treatment Effect on Fatigue in Patients with Ankylosing Spondylitis: A Mediation Analysis. Rheumatol Ther.

[35] Bartlett SJ, Bingham CO, van Vollenhoven R (2022). The impact of tofacitinib on fatigue, sleep, and health-related quality of life in patients with rheumatoid arthritis: a post hoc analysis of data from Phase 3 trials. Arthritis Res & Ther.

[36] Jason LA, Evans M, Brown M (2010). What is fatigue? Pathological and nonpathological fatigue. Prev Med Rep.

[37] Santos EJF, Farisogullari B, Dures E (2023). Efficacy of non-pharmacological interventions: a systematic review informing the 2023 EULAR recommendations for the management of fatigue in people with inflammatory rheumatic and musculoskeletal diseases. RMD Open.

